# Benign Surgery, Malignant Rhythm: Post-laparoscopic Sick Sinus Syndrome in a Child

**DOI:** 10.7759/cureus.104640

**Published:** 2026-03-03

**Authors:** Greeshma Suresh, Sarita Chowdhary, Umang K Agrawal

**Affiliations:** 1 Pediatric Surgery, Institute of Medical Sciences, Banaras Hindu University, Varanasi, IND; 2 General Surgery, Institute of Medical Sciences, Banaras Hindu University, Varanasi, IND

**Keywords:** laparoscopic appendectomy, pediatric surgery, pneumoperitoneum complications, postoperative bradycardia, sick sinus syndrome

## Abstract

Laparoscopic appendectomy is one of the most frequently performed surgical procedures in pediatric surgery and is considered highly safe. Hemodynamic changes produced by pneumoperitoneum are usually transient and clinically insignificant in otherwise healthy children. We report the case of a nine-year-old boy who developed severe postoperative bradycardia and hypotension following an uncomplicated laparoscopic appendectomy. Electrocardiography and echocardiography demonstrated findings suggestive of sick sinus syndrome with transient ventricular dysfunction requiring inotropic support. The child recovered completely within 72 hours with conservative management. This case highlights an uncommon but important cardiovascular complication following pneumoperitoneum and emphasizes the need for vigilant postoperative monitoring even after routine laparoscopic surgery in children.

## Introduction

Laparoscopic appendectomy has become the preferred surgical treatment for acute appendicitis in children because of decreased postoperative pain, earlier ambulation, shorter hospital stay, and superior cosmetic results compared with open surgery. Despite its favorable safety profile, the physiological consequences of carbon dioxide pneumoperitoneum can significantly influence cardiovascular function [1,2]. Creation of pneumoperitoneum increases intra-abdominal pressure, which reduces venous return and cardiac preload while increasing systemic vascular resistance. In addition, vagal stimulation caused by peritoneal stretch may precipitate bradycardia during insufflation [3]. These effects are generally short-lived and resolve after desufflation.

Persistent postoperative arrhythmia, particularly sick sinus syndrome in previously healthy pediatric patients, is extremely rare. Recognition is important because prolonged sinus node dysfunction may lead to severe hemodynamic compromise and can be mistaken for anesthetic drug effects or hypovolemia [4].

We present a rare case of transient sick sinus syndrome occurring after routine laparoscopic appendectomy in a child with no prior cardiac disease.

## Case presentation

A nine-year-old boy presented to the pediatric surgery outpatient department at the Institute of Medical Sciences, Banaras Hindu University, Varanasi, with complaints of abdominal pain for two days. The pain initially began around the umbilicus and later migrated to the right iliac fossa. It was colicky in nature and associated with nausea and low-grade fever that subsided with oral medication. There was no vomiting, abdominal distension, or altered bowel or bladder habits. The child had no known comorbid illnesses and no prior history suggestive of cardiac disease. On examination, the patient was afebrile, with a pulse rate of 96 beats per minute and blood pressure of 80/60 mmHg. Abdominal examination revealed localized tenderness in the right iliac fossa with positive McBurney’s point tenderness.

**Figure 1:**
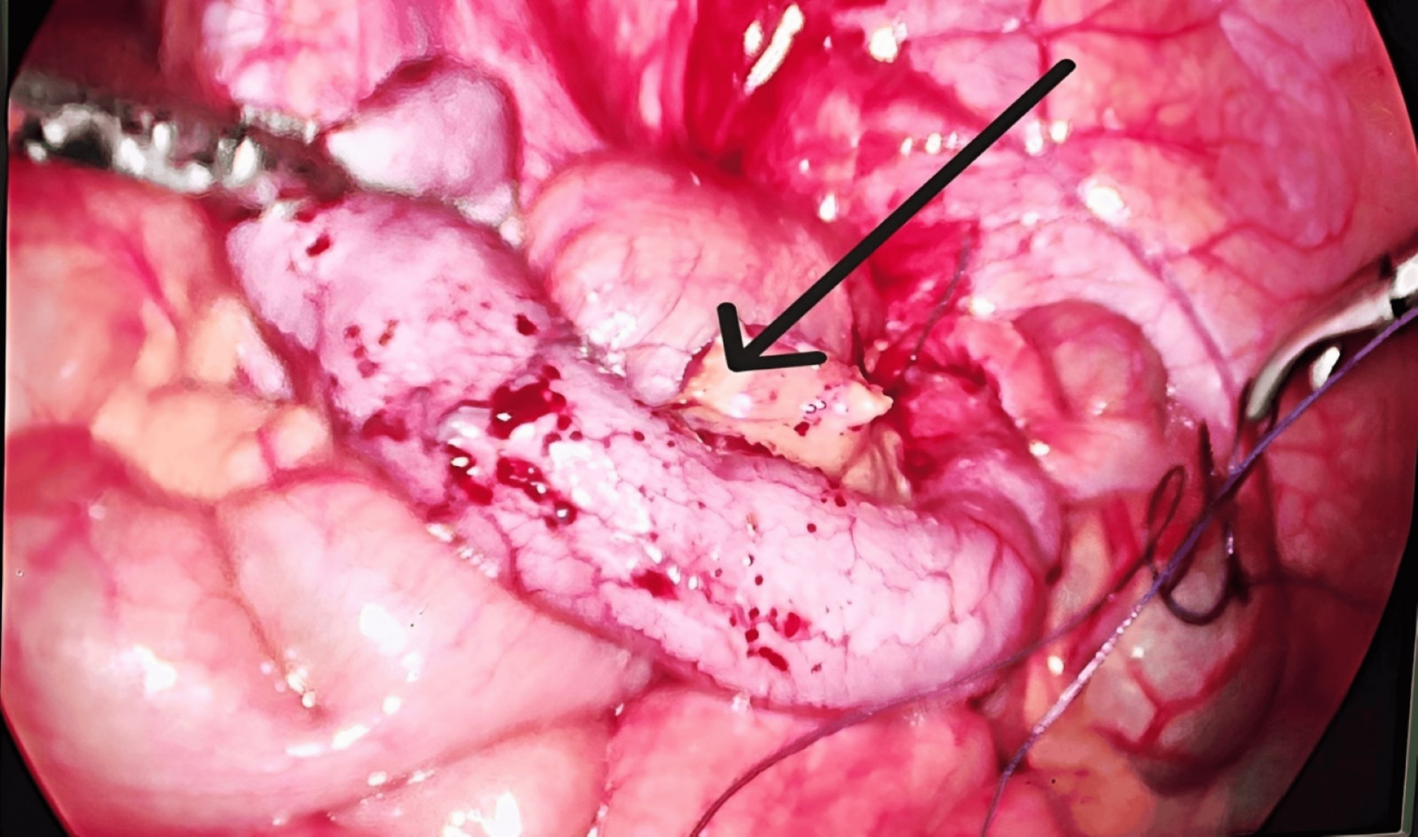
Intraoperative laparoscopic view during appendectomy Laparoscopic visualization of the inflamed appendix with surrounding congested mesoappendix during dissection (black arrow). No evidence of perforation or intra-abdominal contamination was noted intraoperatively. The procedure was completed uneventfully.

Laboratory investigations showed hemoglobin of 11.4 g/dL, total leukocyte count of 16,900/µL, and platelet count of 1.19 lakh/µL. Serum electrolytes were within acceptable limits (sodium 137.2 mmol/L, potassium 3.38 mmol/L, and chloride 92.4 mmol/L). Ultrasonography of the abdomen demonstrated a noncompressible enlarged appendix measuring 13.6 mm, with wall thickening and periappendiceal fat stranding suggestive of acute appendicitis. The child was admitted and underwent standard laparoscopic appendectomy under general anesthesia in the Department of Pediatric Surgery. The procedure was uneventful (Figure 1), and the patient was extubated after surgery.

Postoperative complication

**Figure 2:**
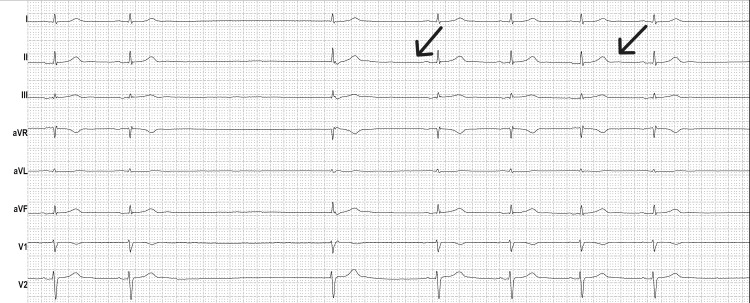
Postoperative electrocardiogram demonstrating sinus bradycardia (black arrows) Electrocardiogram obtained during the hemodynamic instability phase showing sinus rhythm with a decreased heart rate and narrow QRS complexes, consistent with sinus node dysfunction.

In the immediate postoperative period, the patient developed sudden hemodynamic instability characterized by persistent bradycardia, with a heart rate between 56 and 65 beats per minute, and severe hypotension, with blood pressure of 50/40 mmHg. Marked respiratory variation in pulse pressure was also noted. Electrocardiography revealed sinus bradycardia (Figure 2), with narrow QRS complexes consistent with sinus node dysfunction. Two-dimensional echocardiography showed mild left ventricular dysfunction with dilated chambers and grade II diastolic dysfunction. These findings were suggestive of transient sick sinus syndrome with myocardial depression.

Management

The child was immediately shifted to the pediatric intensive care unit due to symptomatic bradycardia with poor perfusion. Initial stabilization included airway support, supplemental oxygen, and evaluation for reversible causes such as hypoxia, hypovolemia, and electrolyte imbalance. Continuous cardiac monitoring confirmed persistent sinus bradycardia associated with hypotension. According to Pediatric Advanced Life Support guidelines, pharmacologic therapy is indicated when bradycardia is associated with poor perfusion or hypotension, and vasoactive support should be initiated promptly [5].

An intravenous epinephrine infusion was started at 0.05 µg/kg/min and titrated according to perfusion parameters and mean arterial pressure. Low-dose epinephrine improved chronotropy and myocardial contractility while also providing vasoconstrictor support. Despite a partial response, blood pressure remained labile, suggesting reduced vascular tone and myocardial dysfunction. A vasopressin infusion was therefore added as a second-line vasopressor to augment systemic vascular resistance and improve coronary perfusion. Fluid administration was carefully optimized to maintain preload while avoiding fluid overload in view of ventricular dysfunction. Urine output, capillary refill, and perfusion parameters were continuously monitored.

Over the next 24-48 hours, gradual improvement in heart rate and blood pressure was observed, with restoration of stable sinus rhythm. The epinephrine infusion was tapered once age-appropriate heart rate and perfusion were achieved, followed by discontinuation of vasopressin. By postoperative day 3, all vasoactive medications were successfully weaned. Enteral feeds were initiated and tolerated well. The child remained stable and was discharged on postoperative day 5 without recurrence of arrhythmia.

## Discussion

Carbon dioxide pneumoperitoneum produces predictable cardiovascular alterations that are usually well tolerated in healthy children. However, the pediatric circulation is uniquely vulnerable because of higher resting vagal tone and reduced cardiovascular reserve. An increase in intra-abdominal pressure reduces venous return by compressing the inferior vena cava, thereby decreasing preload and stroke volume. Simultaneously, hypercarbia and peritoneal stretch trigger parasympathetic activation through vagal afferents, predisposing patients to bradyarrhythmias [1-3].

In the present case, bradycardia persisted beyond the intraoperative period and was accompanied by hypotension and ventricular dysfunction, suggesting pathologic sinus node suppression rather than a transient vagal reflex. Sustained reduction in preload decreases coronary perfusion pressure, particularly affecting the sinoatrial node, which is highly sensitive to perfusion changes. The resulting ischemia-like state produces functional suppression of automaticity resembling sick sinus syndrome [4,6]. Pneumoperitoneum also increases systemic vascular resistance and afterload. The combination of reduced preload and increased afterload results in acute ventricular-arterial uncoupling and transient myocardial dysfunction resembling myocardial stunning. Echocardiographic findings of dilated chambers and diastolic dysfunction in our patient support reversible myocardial depression rather than structural cardiomyopathy [1,7,8].

Children exhibit exaggerated cardiorespiratory interactions; therefore, the marked respiratory pulse pressure variation likely reflected preload dependence and impaired ventricular filling. Rapid improvement following catecholamine therapy supports a functional conduction abnormality rather than intrinsic sinus node disease. Hypercarbia-mediated autonomic imbalance may further contribute to conduction disturbances. Carbon dioxide absorption stimulates both the sympathetic and parasympathetic systems; however, in children, vagal predominance may result in profound bradycardia [7].

The absence of recurrent arrhythmia, normalization of ventricular function, and complete recovery without pacing confirm a reversible pneumoperitoneum-induced autonomic and hemodynamic disturbance. Awareness of this mechanism is important because persistent postoperative bradycardia is often misattributed to anesthetic drugs or hypovolemia, delaying appropriate management.

## Conclusions

Although laparoscopic appendectomy is considered a routine and safe pediatric procedure, pneumoperitoneum can rarely precipitate significant cardiovascular instability. Persistent postoperative bradycardia should not be presumed to be a benign anesthetic or vagal effect and warrants prompt evaluation for underlying conduction disturbance. Transient sick sinus syndrome may occur because of combined autonomic imbalance, reduced preload, and impaired coronary perfusion during laparoscopy.

Early recognition and supportive hemodynamic management can result in complete recovery without the need for pacing or invasive cardiac intervention. This case underscores the importance of vigilant postoperative monitoring in children undergoing laparoscopic surgery and highlights the need for awareness of reversible functional cardiac complications even after uncomplicated procedures.
